# Supplementary material for: Dynamic transcriptomic profiles of zebrafish gills in response to zinc supplementation
Source: BMC Genomics. 2010 Oct 11;11:553. doi: 10.1186/1471-2164-11-553 (PMC3091702; doi:10.1186/1471-2164-11-553)
Supplement: Additional file 2 — Interactive Direct Interaction Network representing the molecular interactions between zinc, copper, iron, calcium and proteins encoded by transcripts changed by zinc supplementation. Mini web-site containing index.html and hyperlinked pages in subdirectory describing a Direct Interaction Network automatically generated based on curated interactions contained within the proprietary PathwayArchitect database. Ovals represent proteins and the circles symbolize metal ions. Objects are coloured by their abundance in zebrafish at the time-point they were significantly different from the control is a scale from -4 fold (dark green) to +4 fold (dark red). Where significant differences were found at more than one time-point, the colour overlay shows expression at the first instance. Dark blue squares denote 'binding', and light blue squares 'expression'; green squares stand for 'regulation', green diamonds for 'metabolism', and green circles for 'promoter binding'. Arrow heads indicate directionality of the interaction where annotated. All nodes and edges can be further interrogated by selecting the relative area of the image. [file 1471-2164-11-553-S2.zip › PathwayArchitect Zn xs DIN/150922.html]

# PROTEIN: SHBG

|  |  |
| --- | --- |
| Name | SHBG |
| Type | PROTEIN |
| Description | sex hormone-binding globulin |
| Note | Plays a role in extracellular transport of androgens [RGD] |
| Alias | ABP |
|  | Abpa |
|  | Sex hormone binding globulin or androgen-binding protein (other gene product from ABP gene) |
|  | Sex hormone-binding globulin (androgen binding protein) |
|  | Sex hormone binding globulin or androgen-binding protein |


---

|  |  |
| --- | --- |
| GO Component | extracellular space |
|  | extracellular region |


---

|  |  |
| --- | --- |
| GO ID | GO:0005615 |
|  | GO:0009914 |
|  | GO:0006520 |
|  | GO:0005497 |
|  | GO:0042803 |
|  | GO:0016831 |
|  | GO:0005515 |
|  | GO:0005576 |
|  | GO:0005496 |


---

|  |  |
| --- | --- |
| MIM | MIM:182205 |


---

|  |  |
| --- | --- |
| Connectivity | 600 |


---

|  |  |
| --- | --- |
| Entrez ID | 24775 |
|  | 20415 |
|  | 6462 |


---

|  |  |
| --- | --- |
| Agilent ID | A\_23\_P207088 |
|  | A\_42\_P573643 |
|  | A\_53\_P148803 |
|  | A\_51\_P423549 |
|  | A\_14\_P135915 |
|  | A\_23\_P207089 |
|  | A\_53\_P121299 |


---

|  |  |
| --- | --- |
| Cellular Localization | Extracellular region |


---

|  |  |
| --- | --- |
| Pathway | Zn xs inventory |
|  | Zn xs DIN |


---

|  |  |
| --- | --- |
| GO Process | amino acid metabolism |
|  | hormone transport |


---

|  |  |
| --- | --- |
| UniGene | Mm.1431 |
|  | Hs.46319 |
|  | Rn.37473 |


---

|  |  |
| --- | --- |
| Affymetrix Probeset ID | 100702\_at |
|  | 1368285\_at |
|  | 1421602\_at |
|  | 215689\_s\_at |
|  | 36328\_at |
|  | Hs.46319.1.S1\_3p\_a\_at |
|  | M31179\_at |
|  | M31651\_at |
|  | U85644\_s\_at |
|  | X16349\_s\_at |


---

|  |  |
| --- | --- |
| GO Function | protein binding |
|  | steroid binding |
|  | androgen binding |
|  | carboxy-lyase activity |
|  | protein homodimerization activity |


---

|  |  |
| --- | --- |
| Nucleotide | X05792 |
|  | M15034 |
|  | X16350 |
|  | NM\_011367 |
|  | X05885 |
|  | U85644 |
|  | M31179 |
|  | AL731687 |
|  | NM\_012650 |
|  | AK010959 |
|  | BC069597 |
|  | X05403 |
|  | M31651 |
|  | X16351 |
|  | M19993 |
|  | X16349 |
|  | NM\_001040 |
|  | BC097336 |


---

|  |  |
| --- | --- |
| Protein | AAC18778 |
|  | CAI52019 |
|  | AAH69597 |
|  | AAA40649 |
|  | CAA34399 |
|  | NP\_035497 |
|  | CAA29309 |
|  | AAB42088 |
|  | AAA40648 |
|  | P08689 |
|  | P04278 |
|  | NP\_036782 |
|  | P97497 |
|  | CAA29234 |
|  | NP\_001031 |
|  | CAA34400 |
|  | CAA34398 |
|  | CAA28987 |
|  | AAH97336 |
|  | AAA40650 |


---

|  |  |
| --- | --- |
| Organism | Mammal |


---

|  |  |
| --- | --- |
| Location | chromosome 17, 17p13-p12 (Homo sapiens) |
|  | 11 35.0 cM (Mus musculus) |
|  | chromosome 10, 10q24 (Rattus norvegicus) |
|  | chromosome 11, 11 35.0 cM, 11 B3 (Mus musculus) |


---

|  |  |
| --- | --- |
